# Supplementary material for: Predicting compound-protein interaction using hierarchical graph convolutional networks
Source: PLoS One. 2022 Jul 21;17(7):e0258628. doi: 10.1371/journal.pone.0258628 (PMC9302762; doi:10.1371/journal.pone.0258628)
Supplement: S1 Appendix — The change in performance of the prediction models when we omit either the compound or protein branch. (PDF) [file pone.0258628.s001.pdf]

Supplementary

1 Experimental result

Table 1 shows the F1 score of DeepDTA and HGCN models on testing data when we omit either the compound or protein branch from the prediction models. The performance of these models drops after the omission. As a result, these models have used the information from both proteins and compounds during their prediction. In the case of chembl27 data, F1 score of HGCN model reduces dramatically, from 0.797 to 0.001, when the model ignores the compound branch. Meanwhile, it reduces only 11.2% when the model ignores the protein branch. This implies that the compounds contribute much more than the proteins into the prediction outcome in general. We also observe this situation in C. elegans and human data but the difference is much smaller. This can be explained by the gap between the compounds and the proteins in the training data. For DeepDTA model, we also encounter the same situation in chembl27 and C.elegans data.

	**DeepDTA**			**HGCN**		
**dataset**	full	no compound	no protein	full	no compound	no protein
C.elegans	0.973	0.760	0.795	0.950	0.634	0.891
human	0.944	0.785	0.753	0.947	0.733	0.854
chembl27	0.751	0.009	0.671	0.797	0.001	0.685

Table 1: F1 score of DeepDTA and HGCN on testing data when omitting either the compound or protein branch from the prediction models.
